# Reassessing the Role of APOBEC3G in Human Immunodeficiency Virus Type 1 Infection of Quiescent CD4+ T-Cells

**DOI:** 10.1371/journal.ppat.1000342

**Published:** 2009-03-20

**Authors:** Masakazu Kamata, Yoshiko Nagaoka, Irvin S. Y. Chen

**Affiliations:** 1 Department of Microbiology, Immunology and Molecular Genetics, David Geffen School of Medicine, University of California Los Angeles, Los Angeles, California, United States of America; 2 Department of Molecular and Medical Pharmacology, David Geffen School of Medicine, University of California Los Angeles, Los Angeles, California, United States of America; Northwestern University, United States of America

## Abstract

HIV-1 is restricted for infection of primary quiescent T-cells. After viral entry, reverse transcription is initiated but is not completed. Various hypotheses have been proposed for this cellular restriction including insufficient nucleotide pools and cellular factors, but none have been confirmed as the primary mechanism for restriction. A recent study by Chiu et al. implicates APOBEC3G, an anti-retroviral cytidine deaminase, as the cellular restriction factor. Here, we attempted to confirm these findings using the same strategy as reported by Chiu et al. of siRNA targeting knock-down of APOBEC3G expression. In contrast to the published study, our results do not support a role for APOBEC3G in restriction of HIV-1 in quiescent CD4+ T-cells. In our study, we tested the same siRNA as reported by Chiu et al. as well as two additional siRNAs targeting APOBEC3G, one of which showed 2-fold greater knock-down of APOBEC3G mRNA. However, none of the three siRNAs tested had a discernable effect on enhancing infection by HIV-1 in quiescent CD4+ T-cells. Therefore, we conclude that the primary mechanism of HIV-1 restriction in quiescent CD4+ T-cells remains to be elucidated.

## Author Summary

In 1990, we demonstrated that unstimulated quiescent T-lymphocytes are resistant to HIV-1 infection. Viruses can get into the cell, and reverse transcription is started, but it is not completed. Various hypotheses have been proposed for this blockage including insufficient free nucleotides and inhibiting cellular factors, but none have been confirmed as the primary mechanism for this blockage. A recent study by Chiu et al. provided one possible mechanism for the blockage in quiescent T-lymphocytes: APOBEC3G, an anti-retroviral cytidine deaminase that was implicated as the responsible factor for this blockage. We have attempted to confirm these findings using published methods. Chiu et al. reported a 37-fold enhancement of HIV-1 infection in quiescent T-lymphocytes by decreasing the levels of APOBEC3G. In contrast to the published study, our results do not support a role for APOBEC3G in the inhibition of HIV-1 infection in quiescent T-lymphocytes. We believe the block of HIV-1 infection of quiescent T-cells remains unknown.

## Introduction

Nonproliferating quiescent CD4+ T-cells are resistant to the infection with human immunodeficiency virus type 1 (HIV-1) unless they are activated by mitogenic stimulation [1]–[5] or by cytokine stimulation [6]. Other studies demonstrated that quiescent T-cells at the stage of G_0_ or G_1a_ of the cell cycle are nonpermissive to HIV-1 infection, but cells in the G_1b_ phase of the cell cycle which show high levels of RNA synthesis, but no DNA synthesis [7] are permissive, and the induction of cell cycle progression per se is not needed to render cell permissive [8],[9]. Earlier studies showed that HIV-1 infection in quiescent CD4+ T-cells results in 4-fold lower levels of viral entry, and incomplete reverse transcription and minimum levels of integration are observed [5],[10],[11]. Although the precise mechanisms involved in this blockage remain unidentified, a number of cellular factors have been reported as restriction factors to HIV-1 infection in quiescent CD4+ T-cells (reviewed in more detail [12],[13]), including Murr1 [14] and most recently APOBEC3G [15].

APOBEC3G is a cytidine deaminase and has well characterized potent anti-retroviral activity, including against HIV-1 [16]–[20]. In the case of HIV-1, it acts through incorporation into virions where it edits newly synthesized viral DNA in the next infection cycle by deaminating dC to dU, resulting in lethal G-to-A hypermutations in the single stranded viral DNA intermediate (reviewed in [16],[21]). The HIV-1 encoded viral infectivity factor (Vif) counteracts the effects of virion incorporated APOBEC3G by mediating its degradation [22]–[24]. A deaminase-independent anti-viral activity has also been identified [15],[25],[26] but the detailed mechanism of action is poorly understood.

Using siRNA mediated knock-down, Chiu et al. concluded that APOBEC3G plays role in HIV-1 restriction in quiescent resting CD4+ T-cells. In those cells, APOBEC3G exists as a low molecular-mass (LMM) ribonucleoprotein complex that inhibits HIV-1 infection prior to reverse transcription probably through its RNA binding activity. In this report, we attempted to reproduce the findings using the identical strategy of siRNA mediated knock-down of APOBEC3G expression as reported by Chiu et al. By nucleofection of the same siRNA as reported by Chiu et al (siA3G_240WT_) as well as two additional siRNAs targeting APOBEC3G, we confirm reduction in APOBEC3G mRNA and protein in quiescent CD4+ T-cells. However, none of the siRNAs resulted in a significant enhancement of HIV-1 infection in those cells. Therefore, we conclude that the role of APOBEC3G in the mechanism of HIV-1 restriction in quiescent CD4+ T-cells is unclear.

## Results

### Synthesized siRNAs can transduce into quiescent CD4+ T-cells with high efficiency by nucleofection

Chiu et al. reported a 37-fold enhancement of HIV-1 infection by an HSA bearing reporter virus in unstimulated quiescent CD4+ T-cells following nucleofection of siRNA directed to APOBEC3G - a level that is nearly comparable to that observed in PHA/IL-2 stimulated cells [15]. We mimicked the experimental conditions of Chiu et al. using the same siRNA (siA3G_240 WT_) and VSV-G pseudotyped HSA reporter virus (NL4-3 HSA R-E- [27]).

**Figure 1:**
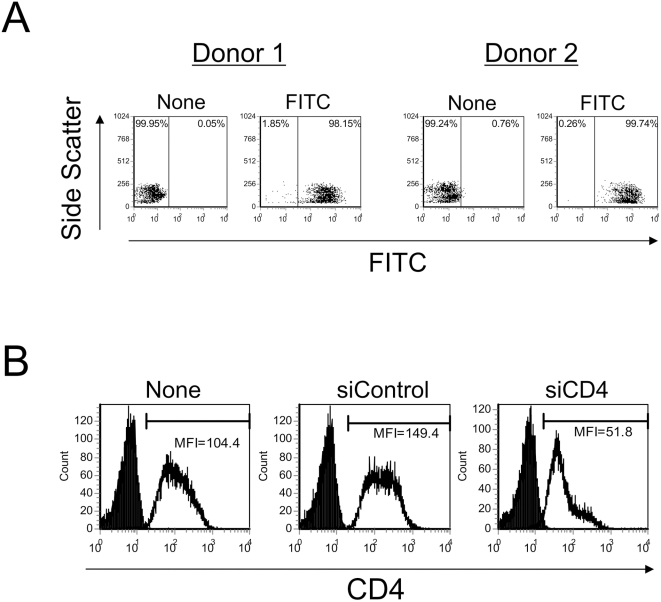
siRNAs can transduce into quiescent CD4+ T-cells with high efficiency by nucleofection. (A) Quiescent CD4+ T-cells were purified from freshly isolated PBMCs of two independent donors with CD4 magnetic beads and nucleofected with siRNA targeting APOBEC3G (siA3G_883_), conjugated with (FITC) or without FITC (None). Cells were analyzed by flow cytometry 3 hr after nucleofection. (B) To monitor the integrity of the RNAi machinery, quiescent CD4+ T-cells derived from PBMCs were nucleofected with siRNA targeting CD4 (siCD4), control siRNA (siControl), or no siRNA (None). The levels of cell surface CD4 expression were monitored by flow cytometry using PE-conjugated anti-CD4 antibody or isotype-matched control 48 hr after nucleofection and represented by mean fluorescent intensity (MFI) in each panel. The solid line represents PE-CD4 antibody stained cells, whereas the shaded area represents isotype control staining.

We first tested the efficiency of nucleofection using fluorescein isothiocyanate (FITC) conjugated siRNA. Quiescent CD4+ T-cells (5×10^6^) were nucleofected with 2 µg of siRNA following the manufacturer's protocol and the efficiency of siRNA transduction monitored by flow cytometry. We consistently observed 5–20% higher mortality between nucleofected cells compared to untreated control cells 48 hr after nucleofection. This level was similar or slightly lower than that reported by Chiu et al. [15]. As shown in Figure 1A, FITC-conjugated siRNA was nucleofected into nearly 100% of the cells from two independent blood donors. We further tested whether the RNAi mechanism is functional in quiescent CD4+ T-cells using siRNA specific to the CD4 molecule. After nucleofection, the cell surface expression of CD4 molecules on quiescent CD4+ T-cells decreased 3-fold as measured by mean fluorescent intensity (MFI) (Figure 1B, siCD4) compared to control siRNA nucleofected cells (Figure 1B, siControl). Identical results were also observed using cells from another independent donor (data not shown).

### The expression of APOBEC3G effectively knock-down by nucleofection of siRNA directed to the sequence of APOBEC3G in quiescent CD4+ T-cells

**Figure 2:**
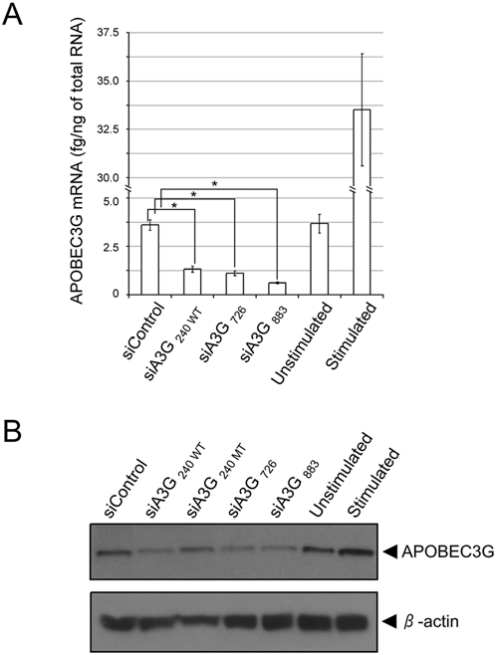
siRNAs directed to the sequence of APOBEC3G effectively knock-down the expression of APOBEC3G in quiescent CD4+ T-cells. (A) Quiescent CD4+ T-cells derived from PBMCs were nucleofected with siRNAs and cultured for two days. Total RNA was isolated, and the levels of APOBEC3G mRNA were monitored by quantitative real time RT-PCR using β-actin as an internal control [31]. P values (asterisks) versus control siRNA were 0.00022 (siA3G_240 WT_), 0.00013 (siA3G_726_), 0.00005 (siA3G_883_), respectively. (B) Quiescent CD4+ T-cells derived from PBMCs were nucleofected with siRNAs and cultured for two days. Cells were lysed in 0.5% SDS, and the levels of APOBEC3G protein were monitored by Western blotting. β-actin was used as a loading control.

We next examined that the levels of knocking-down of APOBEC3G by siRNA reported by Chiu et al., that targeted APOBEC3G (siA3G_240WT_). We also tested two additional siRNAs, one previously published siRNA [28] and another identified by ourselves directed to distinct sequences of APOBEC3G (siA3G_726_ and siA3G_883_, respectively). These siRNAs gave greater downregulation of APOBEC3G mRNA than that reported by Chiu et al.; 6-fold reduction was observed in the case of siA3G_883_ (Figure 2A). We further examined the protein levels of APOBEC3G in siRNA nucleofected cells by Western blotting (Figure 2B). All three siRNAs targeting APOBEC3G showed comparable levels of reduction of APOBEC3G protein at 48 hr after nucleofection; the band intensities of the cells nucleofected with siRNA targeting APOBEC3G compared to that of control siRNA nucleofected cells were 35%, 33%, and 31% by siA3G_240 WT_, siA3G_726_, and siA3G_883_, respectively, a decrease in levels similar to that reported by Chiu et al [15].

### APOBEC3G knock-down in quiescent CD4+ T-cells does not affect HSA reporter virus infection

**Figure 3:**
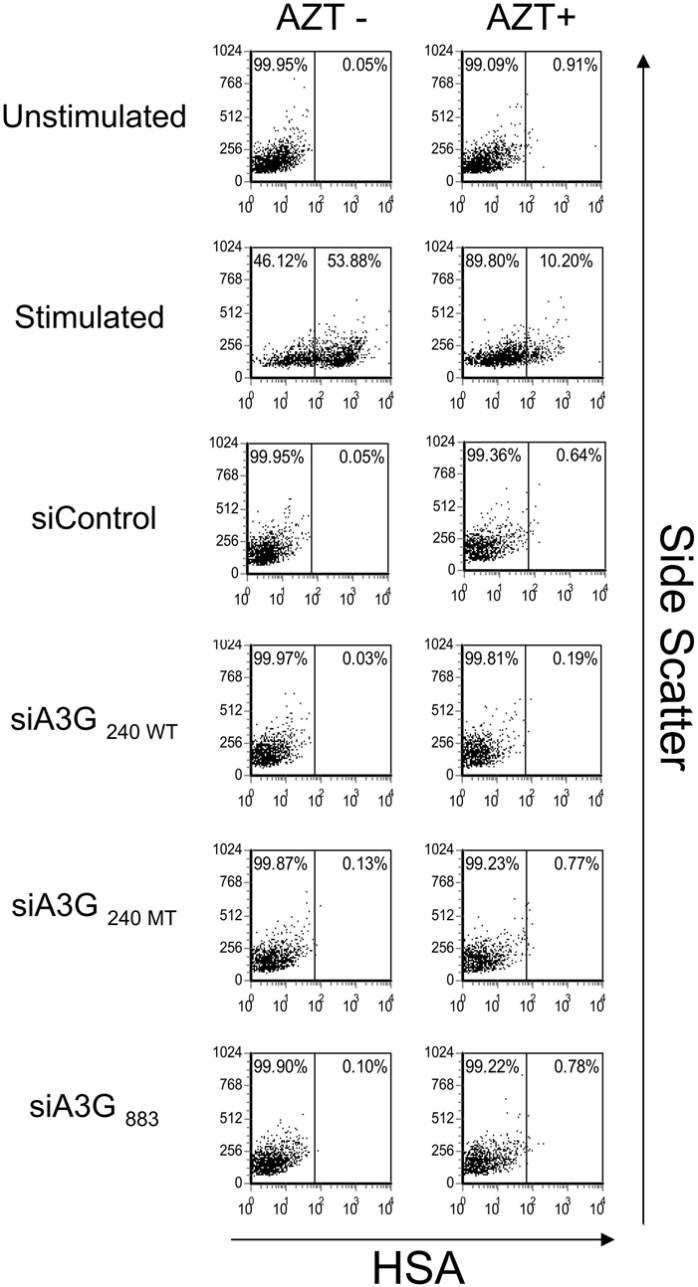
siRNA mediated knock-down of APOBEC3G does not affect the infectivity of HIV-1 reporter virus. Quiescent CD4+ T-cells derived from PBMCs were nucleofected with siRNAs and cultured for two days. Cells were then infected with VSV-G pseudotyped NL4-3 HSA reporter virus (125 ng of p24 per 1×10^5^ cells) for 3 hr and subsequently cultured in the absence or presence of 25 µM AZT. Expression of HSA was monitored 48 hr after the reporter virus infection by flow cytometry using PE-conjugated anti-HSA antibody. Unstimulated non-nucleofected cells (Unstimulated) and unstimulated cells nucleofected with control siRNA (siControl) served as negative controls. Cells stimulated with PHA (5 µg/ml) and IL-2 (20 U/ml) served as positive controls (Stimulated). Comparable results were obtained using cells from six different donors.

**Figure 4:**
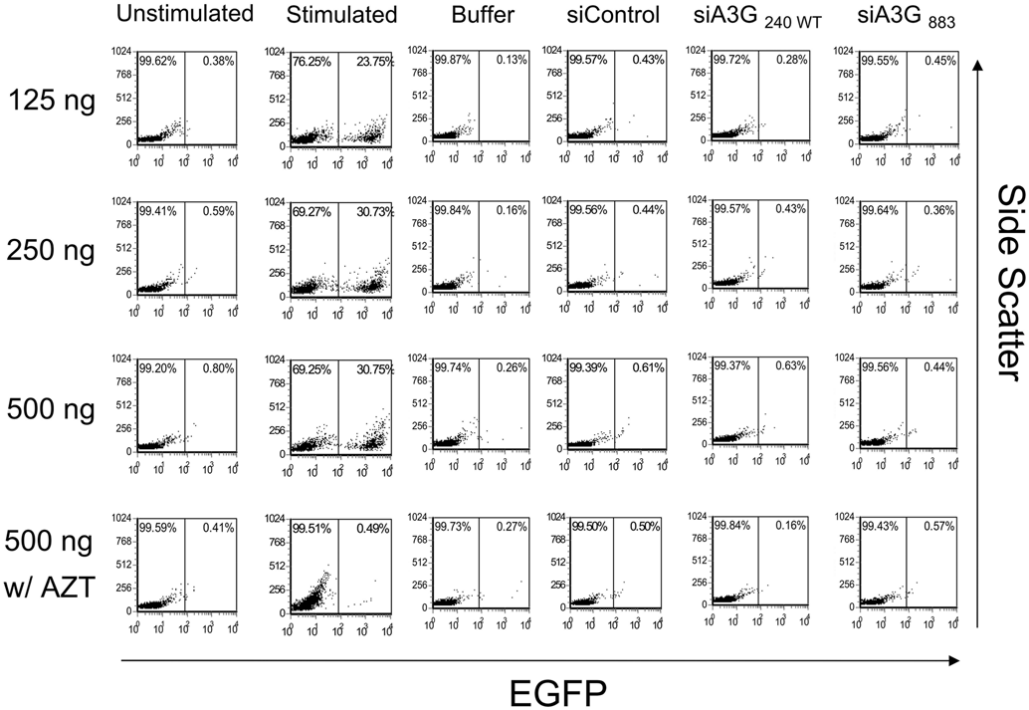
The infectivity of HIV-1 reporter virus on APOBEC3G knocked-down quiescent CD4+ T-cells is not affected by virus multiplicity of infection. Quiescent CD4+ T-cells derived from PBMCs were nucleofected with siRNAs and cultured for two days, three days (Figure S1A), or four days (Figure S1B). Cells were subsequently infected with VSV-G pseudotyped NL4-3 EGFP reporter virus at three different MOIs (125, 250, and 500 ng of p24 per 1×10^5^ cells) for 3 hr, and cultured in the absence or presence of 25 µM AZT. Expression of EGFP was monitored 48 hr after infection by flow cytometry. Unstimulated non-nucleofected cells (Unstimulated) and unstimulated cells nucleofected with control siRNA (siControl) or buffer only (buffer) served as negative controls. Cells stimulated with PHA (5 µg/ml) and IL-2 (20 U/ml) served as positive controls (Stimulated). Comparable results were obtained using cells from four different donors.

**Figure 5:**
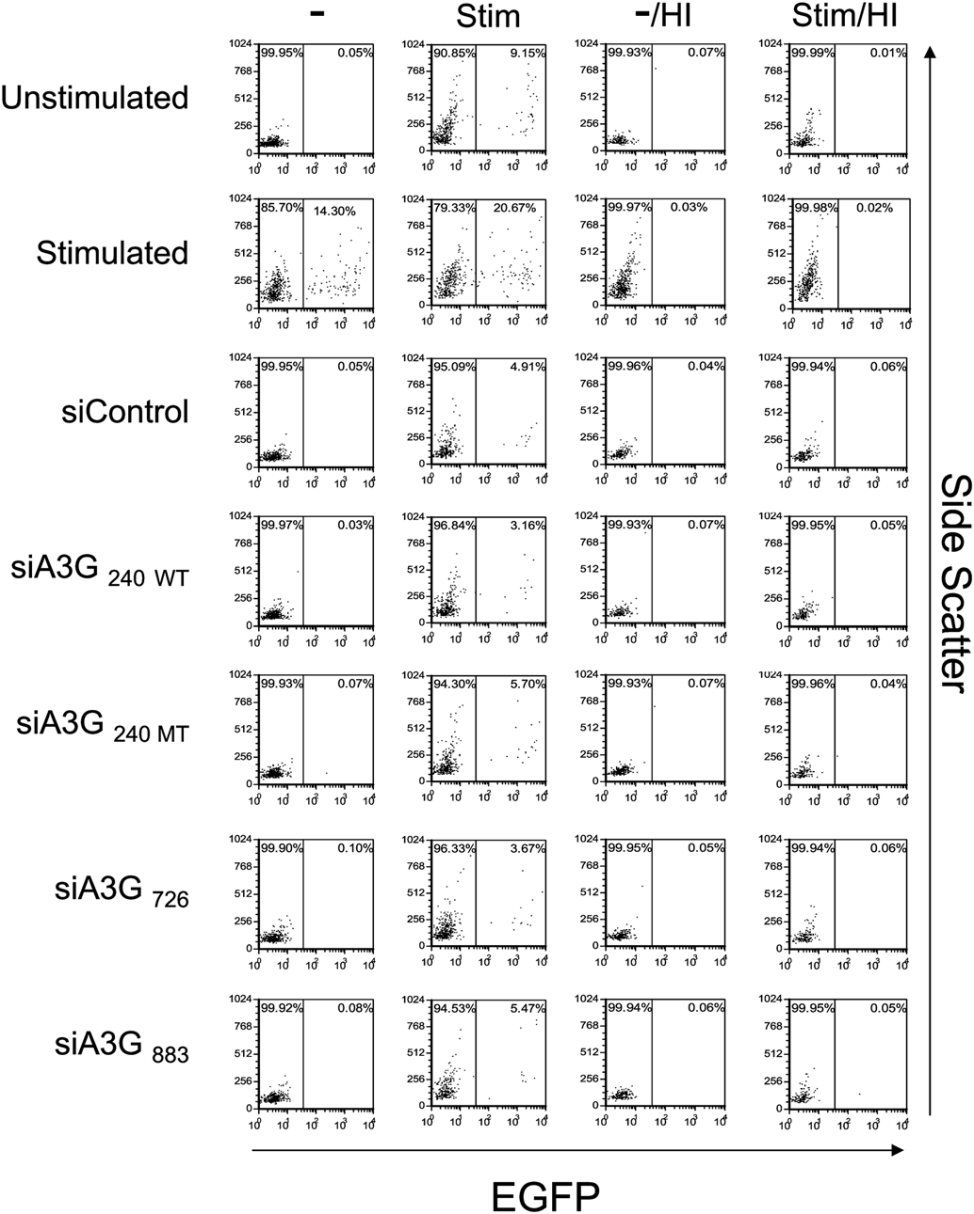
Knock-down of APOBEC3G does not affect the entry of the reporter viruses into quiescent CD4+ T-cells. Quiescent CD4+ T-cells derived from PBMCs were nucleofected with siRNAs and cultured for two days. Cells were then infected with VSV-G–pseudotyped NL4-3 EGFP reporter virus (125 ng of p24 per 1×10^5^ cells) for 3 hr. After infection, half of the cells were stimulated with PHA (5 µg/ml) and IL-2 (20 U/ml) (Stim), and the other half of the cells were cultured without stimulation (−). Expression of EGFP was monitored 48 hr after infection by flow cytometry. Unstimulated non-nucleofected cells (Unstimulated) and unstimulated cells nucleofected with control siRNA (siControl) served as negative controls. Cells stimulated with PHA (5 µg/ml) and IL-2 (20 U/ml) served as positive controls (Stimulated). Comparable results were obtained using cells from three different donors. Heat-inactivated (HI) viruses were used as negative controls for virus infection.

Using above described protocol, we nucleofected siRNAs targeted to APOBEC3G [siA3G_240 WT_, siA3G_726_ (see below), and siA3G_883_] or the mutant (siA3G_240 MT_) and non-specific siRNA (siControl) into quiescent CD4+ T-cells (Figure 3). The cells were then infected by an NL4-3 HSA reporter virus at 2 day post-nucleofection. As expected, CD4+ T-cells stimulated with PHA/IL-2 were highly susceptible to infection by the HSA reporter virus and the virus infection was strongly diminished in the presence of AZT. In striking contrast to Chiu et al., we did not observe any enhancement of HSA expression in quiescent CD4+ T-cells in the presence of siRNA to APOBEC3G reported by Chiu et al. (Figure 3, siA3G_240 WT_). We further tested two additional siRNAs that were more efficient at decreasing APOBEC3G expression as shown in Figure 2. However, there was no discernable effect by these new siRNAs targeting APOBEC3G on enhancing reporter virus infection (Figures 3 and 4, siA3G_883_ are shown; Figure 5, siA3G_726_ and siA3G_883_ are shown). We repeated the experiment using quiescent CD4+ T-cells from 6 different donors and in all cases, we did not observe enhancement of the levels of the HSA expression after nucleofection of APOBEC3G specific siRNA (data not shown). We further tested infection with three different amounts of NL4-3 virus carrying the EGFP reporter rather than the HSA reporter. The infection efficiency reached maximal level with 125 ng of p24 per 1×10^5^ cells (Figure 4). However, we did not observe any significant differences in EGFP expression between the cells nucleofected with siRNAs targeting APOBEC3G (Figure 4, siA3G_240 WT_ and siA3G_883_) or control siRNA (Figure 4, siControl) even with the higher amounts of virus (250 ng and 500 ng of p24). Chiu et al. also reported that the knock-down of APOBEC3G by siRNA in quiescent CD4+ T-cells continued for at least 88 hr after nucleofection [15]. Therefore, we examined infection with the same EGFP reporter virus at 72 and 96 hr after nucleofection (Figures S1A and S1B, respectively). However, we did not observe any effect of APOBEC3G siRNA transduction on EGFP expression at those time points. Furthermore, we tested the same culture medium used by Chiu et al.[15] that contained 10% fetal calf serum (FCS) instead of 10% human serum, but neither culture condition showed any enhancement of reporter virus infection in quiescent CD4+ T-cells (data not shown). Thus, our results obtained under similar conditions were discrepant with those of Chiu et al.

### Knock-down of APOBEC3G does not interfere with HIV-1 entry into quiescent CD4+ T-cells

To ensure that the reporter viruses successfully entered quiescent CD4+ T-cells and to exclude the possibility that the nucleofection prevented the infection of the reporter virus, quiescent CD4+ T-cells were infected with virus and immediately stimulated with PHA/IL2. The marker gene expression was monitored by flow cytometry at 48 hr after stimulation. The stimulation status was monitored by the expression levels of two activation markers, CD25 and CD69. Nucleofection did not affect the expression levels of the activation markers (Figure S2). Similar to the results seen in Figures 3 and 4, we did not detect significant gene expression from the reporter virus in APOBEC3G specific siRNAs nucleofected cells (Figure 5, siA3G_240 WT_/-, siA3G_726_/- and siA3G_883_/-). Upon stimulation by PHA/IL-2, EGFP expression was observed in all nucleofected cells (Figure 5, siA3G_240 WT_/Stim, siA3G_726_/Stim and siA3G_883_/Stim). There was a 2–3 fold difference in EGFP expression between unstimulated control cells (Figure 5, Unstimulated/Stim) and siRNA nucleofected cells (Figure 5, siControl/Stim, siA3G_240 WT_/Stim, siA3G_726_/Stim, and siA3G_883_/Stim). However, there were no obvious differences between the cells nucleofected with siRNAs targeting APOBEC3G (Figure 5, siA3G_240 WT_/Stim, 3.16%; siA3G_726_/Stim, 3.67%; and siA3G_883_/Stim, 5.47%) and the mutant (Figure 5, siA3G_240__MT_/Stim, 5.70%) or control siRNA (siControl/Stim, 4.91%). This result indicated that similar amounts of reporter viruses entered into the quiescent CD4+ T-cells under the five experimental conditions, and none of siRNAs targeting APOBEC3G interfered with the entry of the reporter viruses into quiescent CD4+ T-cells. As described above, the difference in EGFP expression between siRNA nucleofected cells and unstimulated control cells after the stimulation is likely to be caused by the cytotoxic effect of nucleofection. The mortality rate increased when using the combination of nucleofection and post-stimulation (45–50% mortality of siRNA nucleofected cells, compared to 25% in unstimulated control cells). Upon infection with heat inactivated reporter virus (Figure 5, HI), no EGFP expression was observed upon stimulation, indicating that the EGFP signals derived from the infected vectors and were not due to pseudo-infection or auto-fluorescence from the cells. Thus, the knock-down of APOBEC3G by siRNA does not affect the efficiency of HIV-1 entry in quiescent CD4+ T-cells.

### Knock-down of APOBEC3G does not affect the levels of reverse transcription in HIV-1–infected quiescent CD4+ T-cells

**Figure 6:**
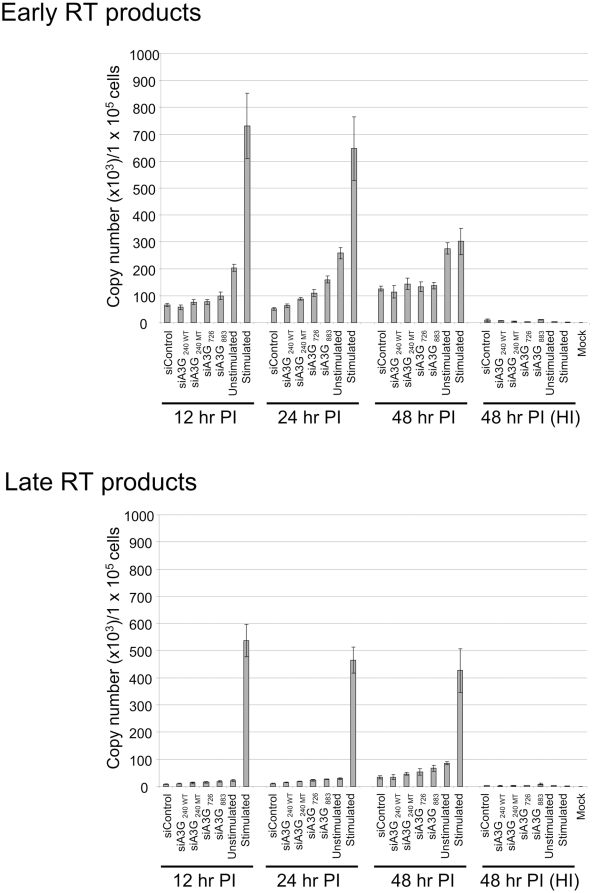
Knock-down of APOBE3G does not affect the levels of reverse transcription in the reporter virus–infected quiescent CD4+ T-cells. Quiescent CD4+ T-cells derived from PBMCs were nucleofected with siRNAs and cultured for two days. Cells were then infected with DNase I–treated VSV-G pseudotyped NL4-3 EGFP reporter virus (125 ng of p24 per 1×10^5^ cells) for 3 hr. Total cellular DNA was isolated at the indicated times and used as a template in quantitative real-time PCR reactions detecting early and late reverse transcripts. Heat-inactivated (HI) viruses were used as negative controls for virus infection. The data represent means±standard deviations calculated from triplicate measurements. Comparable results were obtained from three independent experiments. Mock: untreated control.

HIV-1 can enter into quiescent CD4+ T-cells, but it remains in an inactive state before completion of reverse transcription. Upon subsequent mitogentic stimulation, HIV-1 completes reverse transcription and p24 Gag expression is observable after approximately a 48 hr delay [5],[10]. Chiu et al. reported that this restriction on reverse transcription in quiescent CD4+ T-cells was alleviated by knock-down of APOBEC3G. We lastly examined the status of HIV-1 reverse transcription following downregulation of APOBEC3G. As reported by Vatakis et al.[10], the levels of early reverse transcripts decreased in stimulated cells over time, whereas they hardly changed in unstimulated cells until 48 hr after the reporter virus infection. Similar levels of early reverse transcripts were detected 12 hr after infection and there were no significant differences between siRNAs targeting APOBEC3G (Figure 6, siA3G_240 WT_, siA3G_726_, and siA3G_883_) and control siRNA (Figure 6, siControl) nucleofected cells. Heat inactivation of the reporter viruses for 15 min at 60°C decreased the amount of both early and late reverse transcripts to background levels (Figure 6, HI). The levels of reverse transcription in cells nucleofected with either siRNA targeting APOBEC3G, the mutant, or control siRNA were 2-fold lower than that in the unstimulated control cells (Figure 6, unstimulated). This difference may be caused by the cytotoxic effect of nucleofection as described above; the mortality rate in this experiment was 26–32% in siRNA nucleofected cells 48 hr after nucleofection. In contrast, it was 19% in untreated control cells at the same time point. Comparable results were also observed when we measured late reverse transcripts. The levels of late reverse transcripts increased over time, but the levels were similar or slightly lower than those in unstimulated control cells and, in contrast to the results of Chiu et al. [15], never reached the levels observed in stimulated cells. Equivalent results were obtained when replication competent HIV-1 NL4-3 was used for the infection instead of the reporter virus (data not shown). The above results clearly indicated that the knock-down of APOBEC3G by siRNA affected neither the efficiency of initiation of reverse transcription nor the subsequent elongation of cDNA in quiescent CD4+ T-cells.

## Discussion

The mechanisms involved in the restriction of HIV-1 infection in quiescent CD4+ T-cells remain unidentified. Some potential mechanisms for this blockage may be the presence of cellular inhibitors in quiescent cells [14],[15], the lack of cellular factors required for completion of HIV-1 infection [9] or both. Chiu et al. provided one possible mechanism for the restriction in quiescent T cells [15]. In contrast to their study, our results do not support a role for APOBEC3G in restricting HIV-1 infection of quiescent unstimulated human CD4+ T-cells. We also tested two additional siRNAs that were more efficient at decreasing APOBEC3G, both at the level of mRNA and protein. None of the siRNAs tested resulted in efficient HIV-1 reporter virus infection of quiescent CD4+ T-cells.

It is unclear to us why our results are discrepant with those of Chiu et al. Our experimental conditions for maintaining quiescent T-cells are that which we used previously including in our first description of the block to HIV-1 infection [5]. From our past experience in working with quiescent T-cells, several factors may influence their behavior, including the source of cells and culture conditions. Our cells are obtained from fresh leukopaks from the UCLA blood bank and processed for peripheral blood mononuclear cell (PBMC) by a Ficoll-Hypaque density gradient within 24 hours. The cells are then used within 0.5 hours. In the past we used selected lots of human AB serum monitored for absence of growth stimulating activity rather than FCS. However, if carefully screened, FCS is also satisfactory and we observed no difference between use of 10% FCS and 10% human AB serum in our results (data not shown). Chiu et al. used 10% FCS for the culture of quiescent CD4+ T-cells. We also considered whether the nucleofection method might affect the results, in particular, the cytotoxicity associated with the procedure. Even under optimized conditions, the nucleofected cells consistently contained around 20% dead cells monitored by forward and side scatter compared to untreated control cells 48 hr after nucleofection. Those dead cells might have caused undesired activation of the remaining cells. We monitored the activation status of the nucleofected cells by CD25 and CD69 staining 48 hr after nucleofection, and as Chiu et al. also reported, we did not observe any induction of CD25 and CD69 expression. However, the activation state of T-cell permissive for infection varies depending on the stimuli [6],[8],[9]. Although the conditions reported by Chiu et al., to culture quiescent cells appear to be the same as ours, it is possible that unknown differences in culture conditions may result in subtle differences in cell activation, giving divergent results.

Chiu et al. monitored HIV-1 entry and expression using flow cytometric analysis of HSA reporter gene expression. To exclude any confounding effects of marker gene expression or detection methods, we monitored HIV-1 entry by EGFP expression in addition to HSA expression. However, both detection methods showed identical results - we found no enhancement of HIV-1 entry by knocking-down of APOBEC3G in quiescent CD4+ T-cells.

In conclusion, we performed all our experiments under previously utilized optimum culture conditions to maintain quiescent T-cells and with experimental manipulation identical to Chiu et al., yet, we could not reproduce their results. As such, we believe the mechanism of HIV-1 restriction for quiescent CD4+ T-cells remains to be elucidated.

## Materials and Methods

### Antibodies

PE conjugated anti-mouse CD24, Tri-color conjugated anti-human CD25 and PE conjugated anti-human CD69 monoclonal antibodies and isotype controls were purchased from BD Biosciences (San Jose, CA). Anti-APOBEC3G antibody was obtained from the AIDS Research and Reference Reagent Program at NIH (Cat. No. 10201).

### Primary CD4+ T-cells

Quiescent CD4+ T-cells from fresh human PBMCs were isolated with CD4+ microbeads (Miltenyi Biotec Inc., Auburn, CA) and maintained in hTC medium (Lonza, Rockland, ME) supplemented with 2 mM L-Glutamine and 10% heat-inactivated FCS or human AB serum. For stimulation, isolated CD4+ T-cells were incubated with PHA (Sigma-Aldrich, St. Louis, MO, 5 µg/ml; ) and IL-2 (Roche Diagnostics, Indianapolis, IN, 20 U/ml) for 36 hr followed by IL-2 (20 U/ml) for 12 hr.

### Nucleofection of siRNA

Isolated CD4+ T-cells were transfected with siRNAs using an Amaxa nucleofector (program V-024 as recommended by the manufacturer's protocol for primary human unstimulated T-cells; 2 µg of siRNA per 5–10×10^6^ cells). siRNAs targeting APOBEC3G messenger RNA (Genebank accession number: NM_021822) at residues 240–258 in cording sequence for APOBEC3G (siA3G_240 WT_), 726–746 in cording sequence for APOBEC3G (siA3G_726_), 883–901 in cording sequence for APOBEC3G (siA3G_883_) were chemically synthesized by Qiagen (Chatsworth, CA) or Dharmacon (Chicago, IL). siRNA targeting CD4 (siCD4: #1024675) and control siRNA (siControl: #1027310) were purchased from Qiagen. Since dead cells have lower-forward scatter and higher-side scatter than live cells, cytotoxicity by nucleofection was monitored by measuring the dead cells with flow cytometry [29].

### Virus production and titration

We generated lentiviral vector stocks using an HIV-1 based reporter virus encoding HSA or EGFP (NL4-3 HSA R-E- [27] or NL4-3 EGFP R-E- substituted HSA with EGFP, respectively), packaging plasmid pCMV R8.2 δVpr, and the VSV-G envelope protein-coding plasmid by calcium phosphate-mediated transient transfection as previously described [30]. After 48 and 72 hr, lentiviral vector particles were harvested and concentrated by ultracentrifugation through a 10% sucrose cushion in Hanks' balanced salt solution (HBSS) with 1 mM EDTA and resuspended in a 100-fold lower volume of HBSS and stored at −80°C. The viral titer was measured by anti-p24 Gag ELISA and the infectious titer was determined in 293T cells by infecting with HSA or EGFP encoding vector and flow cytometric analysis.

### Viral infection and detection

Forty-eight hr after nucleofection, CD4+ T-cells were incubated with reporter virus (125 ng of p24 per 1×10^5^ cells) with 8 µg/ml of polybrene. After 3 hr incubation at 37°C, cells were washed extensively with phosphate buffered saline (PBS) and cultured for 48 hr in the presence or absence of 25 µM AZT (A2169; Sigma-Aldrich). Unstimulated and non-nucleofected cells served as negative controls. Reporter gene expression (HSA and EGFP) and the activation markers (CD25 and CD69) were monitored by flow cytometry. Data were collected on a Cytomics FC500 (Beckman Coulter, Fullerton, CA) and analyzed using FCS express (De Novo Software, Los Angeles, CA).

### Real-time PCR

All real-Time PCR quantitations were performed using the BIO-RAD iQ5 system (BioRad, Hercules, CA) in parallel with a set of known quantitative standards. For quantitation of APOBEC3G mRNA, total RNA was extracted from approximately 5×10^5^ cells with TRIZOL and used for quantitative real-time RT-PCR. The iScript one-step RT-PCR kit for probes (BioRad) was used with a 40 ng of total RNA for amplification of APOBEC3G and β-actin as control. The primers used were as follows. APOBEC3G: forward 5′CGCAGCCTGTGTCAGAAAAG3′; reverse, 5′CCAACAGTGCTGAAATTCGTCATA3′; probe, FAM-5′GTGCCACCATGAAGA3′-BHQ1 [31]. β-actin: forward 5′CGAGCGCGGCTACAGCTT3′; reverse, 5′ CCTTAATGTCACGCACGATT3′; probe, HEX-5′ACCACCACGGCCGAGCGG3′-BHQ2. All primers and probe were synthesized by Biosearch Technologies Inc. (Novato, CA). All RT-PCR reactions were carried out as follows: reverse transcription at 50°C for 10 min, inactivation of reverse transcriptase at 95°C for 5 min, and subsequently 45 cycles in two phases consisting of 95°C for 15 sec, and 58°C for 30 sec. APOBEC3G mRNA was normalized using the endogenous β-actin mRNA as a reference.

Virus infection was measured by quantifying HIV-1 early and late reverse transcripts using TaqMan real-time DNA PCR as previously described [5],[8]. Briefly, DNA was extracted from approximately 5×10^5^ cells with urea lysis buffer [4.7 M urea, 1.3% W/V SDS, 0.23 M NaCl, 0.67 mM EDTA, and 6.7 mM Tris-HCl (pH 8.0)] and then subjected to phenol-chloroform extraction and ethanol precipitation. Quantitative real-time DNA PCR was performed by iQ Supermix (BioRad) using primers specific for HIV-1 sequences as previously described [5]. The primer pairs M667/AA55 (R/U5 region) and M667/M661 (LTR/gag region) were used to detect early and late reverse transcripts of HIV-1, respectively. The standard curve used to determine HIV-DNA levels range from 1–1,000,000 copies of NL4-3 DNA. There was no background contamination from DNA of mock infected cells. The amounts of early and late reverse transcripts were normalized using the endogenous β-globin gene as a reference.

### Western blotting for APOBEC3G proteins

1×10^6^ cells were lysed with 0.5% SDS containing protease inhibitor cocktail (P8340; Sigma-Aldrich) and quantified with a BCA protein assay reagent (BioRad). Western blotting was performed as described previously [30]. Briefly, 2.5 µg protein was electrophoresis on 4–20% Precast SDS-PAGE gel (Lonza) and transferred onto immobilon membranes (Millipore, Bedford, MA). After blocking with 5% skim milk in PBS with 0.05% Tween-20 (PBS-T), membranes were reacted with either polyclonal anti-APOBEC3G antibody (Cat. No. 10201, NIH AIDS Research and Reference Reagent Program) or polyclonal anti-β-Actin (Rockland Immunochemicals Inc., Gilbertsville, PA). Membranes were washed with PBS-T three times, treated with secondary antibody conjugated with horseradish peroxidase (Pierce, Rockford, IL) and visualized by chemiluminescence (ECL plus; Amersham Biosciences, Piscataway, NJ).

## Supporting Information

Figure S1 The infectivity of HIV-1 reporter virus on APOBEC3G knocked-down quiescent CD4+ T-cells is not affected by virus multiplicity of infection. Quiescent CD4+ T-cells derived from PBMCs were nucleofected with siRNAs and cultured for three days (A) or four days (B). Cells were subsequently infected with VSV-G pseudotyped NL4-3 EGFP reporter virus at three different MOIs (125, 250, and 500 ng of p24 per 1×10^5^ cells) for 3 hr, and cultured in the absence or presence of 25 µM AZT. Expression of EGFP was monitored 48 hr after infection by flow cytometry. Unstimulated non-nucleofected cells (Unstimulated) and unstimulated cells nucleofected with control siRNA (siControl) or buffer only (buffer) served as negative controls. Cells stimulated with PHA (5 µg/ml) and IL-2 (20 U/ml) served as positive controls (Stimulated). Comparable results were obtained using cells from two different donors.
(5.56 MB TIF)

Figure S2 Knock-down of APOBEC3G does not affect the activation status of quiescent CD4+ T-cells. Quiescent CD4+ T-cells derived from PBMCs were nucleofected with siRNAs and cultured for two days. Cells were then infected with VSV-G pseudotyped NL4-3 EGFP reporter virus (125 ng of p24 per 1×10^5^ cells) for 3 hr. After infection, half of cells were stimulated with PHA (5 µg/ml) and IL-2 (20 U/ml) (Stim), and the other half of the cells were cultured without stimulation (-). The levels of CD25 and CD69 were monitored two days after infection by flow cytometry. Unstimulated none-nucleofected cells (Unstimulated), and unstimulated cell nucleofected with control siRNA (siControl) served as negative controls. Cells stimulated with PHA (5 µg/ml) and IL-2 (20 U/ml) served as positive controls (Stimulated). Comparable results were obtained using cells from three different donors. Heat-inactivated (HI) viruses were used as negative controls for virus infection.
(6.41 MB TIF)
