# Supplementary figures and images for: Reassessing the Role of APOBEC3G in Human Immunodeficiency Virus Type 1 Infection of Quiescent CD4+ T-Cells
Source: PLoS Pathog. 2009 Mar 20;5(3):e1000342. doi: 10.1371/journal.ppat.1000342 (PMC2652112; doi:10.1371/journal.ppat.1000342)

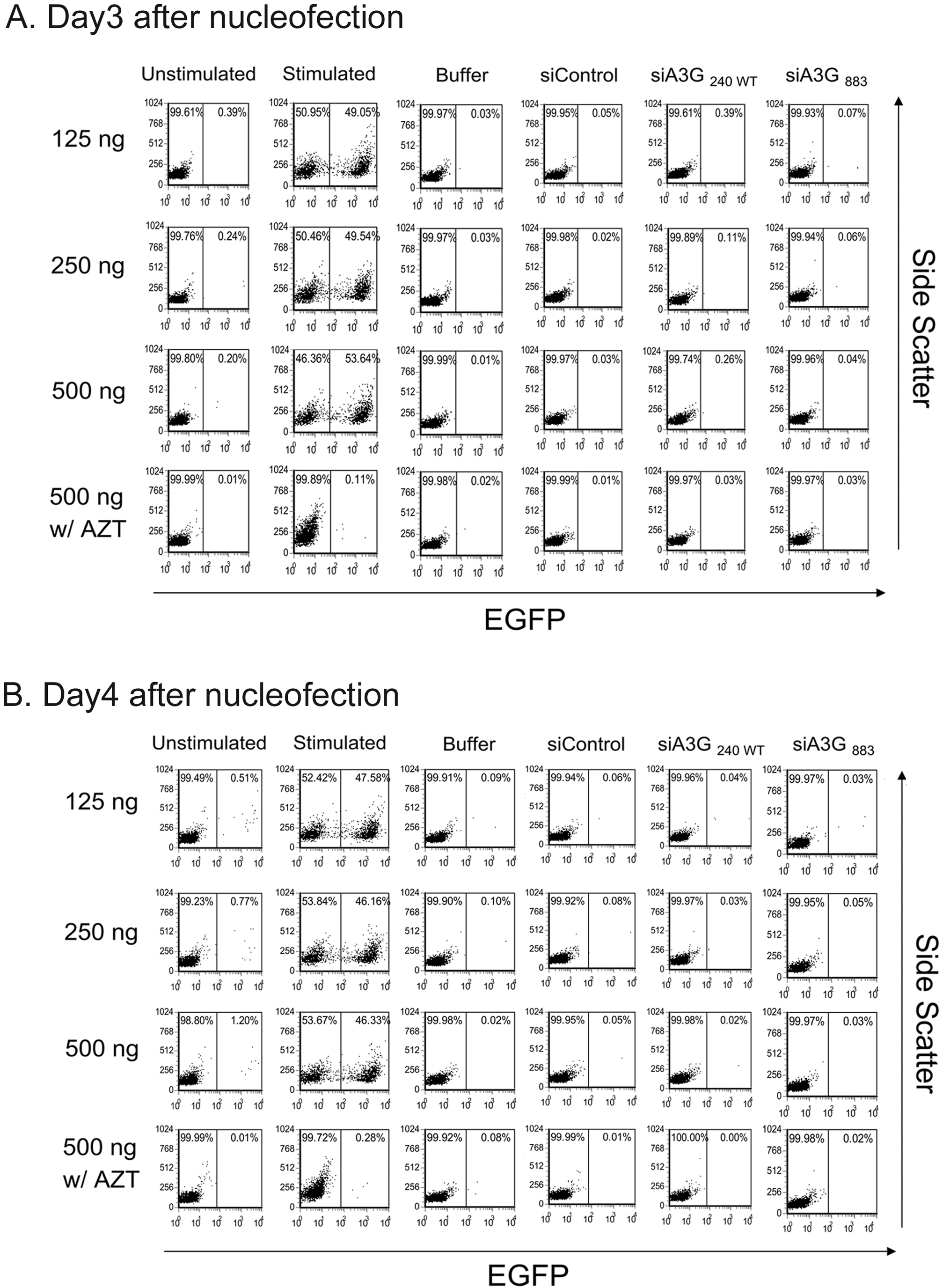

Supplement: Figure S1 — The infectivity of HIV-1 reporter virus on APOBEC3G knocked-down quiescent CD4+ T-cells is not affected by virus multiplicity of infection. Quiescent CD4+ T-cells derived from PBMCs were nucleofected with siRNAs and cultured for three days (A) or four days (B). Cells were subsequently infected with VSV-G pseudotyped NL4-3 EGFP reporter virus at three different MOIs (125, 250, and 500 ng of p24 per 1×10^5^ cells) for 3 hr, and cultured in the absence or presence of 25 µM AZT. Expression of EGFP was monitored 48 hr after infection by flow cytometry. Unstimulated non-nucleofected cells (Unstimulated) and unstimulated cells nucleofected with control siRNA (siControl) or buffer only (buffer) served as negative controls. Cells stimulated with PHA (5 µg/ml) and IL-2 (20 U/ml) served as positive controls (Stimulated). Comparable results were obtained using cells from two different donors. (5.56 MB TIF) [file ppat.1000342.s001.tif]

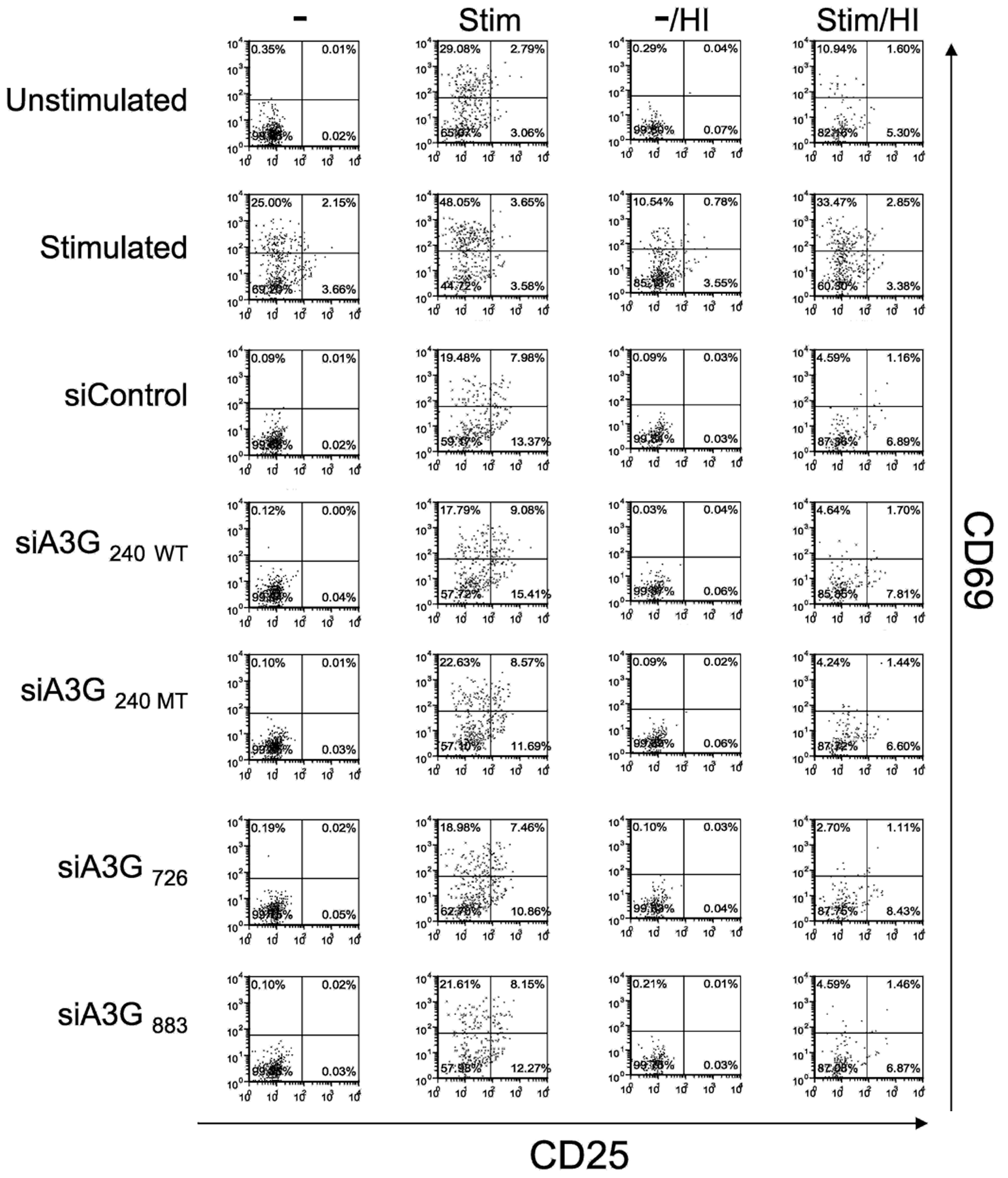

Supplement: Figure S2 — Knock-down of APOBEC3G does not affect the activation status of quiescent CD4+ T-cells. Quiescent CD4+ T-cells derived from PBMCs were nucleofected with siRNAs and cultured for two days. Cells were then infected with VSV-G pseudotyped NL4-3 EGFP reporter virus (125 ng of p24 per 1×10^5^ cells) for 3 hr. After infection, half of cells were stimulated with PHA (5 µg/ml) and IL-2 (20 U/ml) (Stim), and the other half of the cells were cultured without stimulation (-). The levels of CD25 and CD69 were monitored two days after infection by flow cytometry. Unstimulated none-nucleofected cells (Unstimulated), and unstimulated cell nucleofected with control siRNA (siControl) served as negative controls. Cells stimulated with PHA (5 µg/ml) and IL-2 (20 U/ml) served as positive controls (Stimulated). Comparable results were obtained using cells from three different donors. Heat-inactivated (HI) viruses were used as negative controls for virus infection. (6.41 MB TIF) [file ppat.1000342.s002.tif]
